# Supplementary material for: pH-Responsive Succinoglycan-Carboxymethyl Cellulose Hydrogels with Highly Improved Mechanical Strength for Controlled Drug Delivery Systems
Source: Polymers (Basel). 2021 Sep 21;13(18):3197. doi: 10.3390/polym13183197 (PMC8467855; doi:10.3390/polym13183197)
Supplement: Supplementary file 1 [file polymers-13-03197-s001.zip › polymers-1382072-supplementary.pdf]

polymers MDPI

Supplementary Materials

Table of Contents

1. Energy-Dispersive Spectroscopy (EDS) Analysis……..………………………………… p2

2. Summarizing the results obtained in this work with other previously published papers…p3

1. Energy-Dispersive Spectroscopy (EDS) Analysis

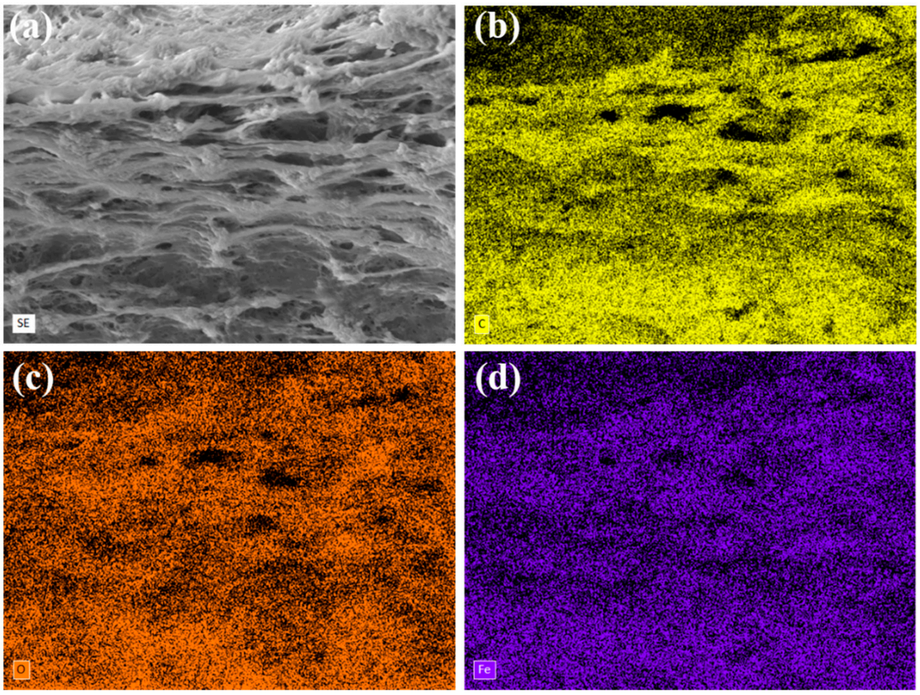

Figure S1. (a) SEM image of S1C1 gel, (b), (c) and (d) EDS mapping image for C, O and Fe

2. Summarizing the results obtained in this work with other previously published papers

	**Acrylic acid /CMC**	**XG[1] /CMC**	**HA[2] /CMC**	**Alg[3] /CMC**	**SG /CMC**
**Gel Characteristics**	Non-bead form Using synthetic polymers	Bead form Using modified natural polymers	Non-bead form Using modified natural polymers	Bead form Using natural polymers	Non-bead form Using natural polymers
**Compressive test (Mechanical strength)**	○	-	○	-	○
**TGA[4]/DTG[5] (Thermal stability)**	-	○	○	-	○
**pH-dependent drug release**	-	○	-	○	○
**Reference**	[27]	[32]	[29]	[30]	-

1: Xanthan Gum 2: Hyaluronic acid 3: Alginate 4: Thermogravimetric analysis 5: Derivative thermogravimetry

Table S1. Summary of gel characteristics, compressive test, TGA/DTG, and pH-dependent drug release results obtained in this study with other previously published papers
